# Validity and Reliability of the Hindi Version of the Modified Child Perceptions Questionnaire 11 to 14

**DOI:** 10.5005/jp-journals-10005-1525

**Published:** 2018-08-01

**Authors:** Prasanna Kumar, Dempsy CM Mandanna, Sanjay M Londhe, Mohit Sharma

**Affiliations:** 1 Professor, Department of Dental Surgery and Oral Health Sciences, Armed Forces Medical College, Pune, Maharashtra, India; 2 Assistant Professor, Department of Pedodontics, Armed Dental Centre (R&R), New Delhi, India; 3 Professor, Department of Orthodontics, Armed Forces Medical College Pune, Maharashtra, India; 4 Associate Professor, Department of Orthodontics, Armed Forces Medical College Pune, Maharashtra, India

**Keywords:** Child perceptions questionnaire, Hindi version of child perceptions questionnaire 11-14, Validity and reliability of child perceptions questionnaire 11-14.

## Abstract

**Introduction:**

The study was conceived to formulate a tool to evaluate child perceptions related to oral health in 11- to 14-year-olds tailor-made for Indian children in Hindi. The original child perceptions questionnaire (CPQ_11-14_) was translated into Hindi and it was tested for validity and reliability.

**Materials and methods:**

The original CPQ_11-14_ was translated into Hindi and some questions were rephrased to suit the sociocultural situation in India. The domains of self-esteem and psychological well-being were added to the questionnaire to broaden the scope of parameters to thoroughly assess the impact on child perceptions toward oral health. The English and Hindi versions of the CPQ_11-14_ were administered during the first visit to test for validity and the children were recalled after 1 week and administered the Hindi questionnaire again after 1 week to check for reliability.

**Results:**

The results showed significant positive correlation between oral symptoms, decayed, missing and filled teeth (DMFT) functional limitation and malocclusion. The mean functional limitation score was found to be higher in subjects with malocclusion in both the English and Hindi questionnaires. The test-retest samples were evaluated using the paired t-test and showed no significant difference between the first and second administration which suggested good reliability.

**Conclusion:**

The translated and modified Hindi CPQ_11-14_ was found to be valid and highly reliable for use in India. The adaptation of the original questionnaire by modifying certain questions to suit the Indian condition was found to be culturally relevant.

**How to cite this article:** Kumar P, Mandanna DCM, Londhe SM, Sharma M. Validity and Reliability of the Hindi Version of the Modified Child Perceptions Questionnaire 11 to 14. Int J Clin Pediatr Dent 2018;11(4):271-276.

## INTRODUCTION

The general health of an individual is influenced by the state of the oral health, thus impacting the overall quality of life (QoL).^1^ This has been brought out aptly in the definition of health given by the World Health Organization, which defines health as a state of complete physical, social and mental well-being. The QoL is defined as an individual’s perception of their position in life in the context of the culture and value system where they live and in relation to their goals, expectations, standards and concerns with regard to their health.^2,3^

Oral health-related quality of life (OHRQoL) is a measure which helps in assessing the functional and psychological impact of oral diseases on individuals. The importance of these tools can be appreciated while evaluating oral health of individuals and communities, making clinical decisions or evaluating success of interventions and also to assess oral health programs and services.^4,5^ Therefore, measurement of OHRQoL can be an important tool for use in community-based oral health surveys.^6,7^

Children are affected by various disorders in the oral and orofacial region, such as malocclusion and dental caries which can potentially compromise social, emotional well-being, and function impacting the OHRQoL.^8,9^ These disorders of orofacial function can affect vital functions, such as breathing, chewing, swallowing, and muscle posture which are important for proper speech, communication and facial expression impacting the perception of OHRQoL.^10,11^ However, there are very few age-specific tools for measuring OHRQoL, especially in children and most of them are in English. This makes it unsuitable for use in populations where English is not spoken. The CPQ is an age-specific validated self-administered questionnaire for measuring OHRQoL developed originally in Canada in English.^12,13^ The CPQs evaluate OHRQoL in four domain subscales of oral symptoms, functional limitations, emotional well-being, and social well-being. The aim of this study was to translate the English version of the CPQ_11-14_ into Hindi to culturally adapt it to the Indian sociocultural situation and evaluate its comprehensibility, validity, and reliability.

## MATERIALS AND METHODS

The testing of the Hindi version of the CPQ_11-14_ was carried out on 40 children who came to our institute along with their parents for the first time seeking orthodontic treatment.

### Inclusion Criteria

- 11- to 14-year-old children
- Understanding/speaking Hindi and English
- No cognitive impairment
- Informed consent from parent
- Assent from child

The CPQ_11-14_ contained questions on various domains; the first two collected demographic details (sex and date of birth). The third question was based on the condition of oral health which was rated according to the Likert scale with five choices ranging from excellent, very good, good, acceptable, or bad. The fourth question related to the extent to which the oral health affected the overall well-being, with choices ranging between not at all, very little, somewhat, a lot, or very much. The responses were scored on a scale of 0 to 4.

The remaining questions were divided into four oral health-related domains, namely oral symptoms (n = 6); functional limitations (n = 9); emotional well-being (n = 9); social well-being (n = 8); self-esteem (n = 14); and psychological well-being (n = 16). Each question had five responses, namely never, once or twice, sometimes, often, and everyday or almost every day with scores ranging from 0 to 4. The questionnaire contained a total of 62 questions and since they were scored based on scores assigned on the Likert scale, the highest possible score was 248 and the lowest, 0. The scores for each subscale were calculated by adding the response scores for particular domain.

The institutional ethical committee clearance was obtained prior to the commencement of the study to rule out any ethical issues. An informed consent form was signed by the parents after they were explained the procedure of the study. Each child participating in the study was explained the procedure and his assent was taken.

A total of 40 questions was administered to the children who had knowledge of both English and Hindi and they were asked to complete the questionnaire without their parent’s assistance. If a child was found taking the help of his parent, that questionnaire was discarded from the study. The child had to fill both the English and Hindi version of the questionnaire during the first visit to the clinic. The child was also clinically examined during this visit to record the caries status using the DMFT index. This index records the scores for each child depending on the number of teeth decayed or filled and missing due to caries. The teeth extracted for other reasons or missing congenitally, unerupted, or lost as a result of trauma are not included. The malocclusion status was also recorded and scored as follows: (0) No malocclusion, (1) slight or moderate, and (2) moderate to severe.

Since the children had visited the clinic along with their parents with the intention of undergoing orthodontic treatment, they were recalled after 1 week and readmin-istered the Hindi version of the questionnaire to check for its reliability. The questionnaires with any question unanswered were excluded from the study and not sent for statistical analysis. Therefore, 7 questions which were incomplete had to be rejected, thus bringing the final sample to 33.

## RESULTS

The total number of children who completed the questionnaire and also successfully completed the retest Hindi questionnaire the second time were n = 33, of which the total no of male children were n = 18 (54.54%) and female children were n = 15 (45.45%). The clinical data showed that 72.7% of these children had some form of oral symptoms (DMFT), 45.4% children had slight-to-moderate malocclusion, and 33.3% had severe malocclu-sion requiring active treatment. The statistical analysis of the results from all the questionnaires was carried out by testing the discriminant validity by first comparing total and subscale scores of the children with relation to their DMFT status and malocclusion scores. The total and subscale scores were calculated for the whole sample using the Spearman’s rank correlation coefficient. The results of the discriminant validity testing for the English questionnaire showed significant positive correlation between oral symptoms, DMFT, and malocclusion. There was also significant positive correlation between functional limitation and malocclusion. The overall total score showed significant positive correlation with malocclusion (Table 1). The discriminant validity testing of the Hindi questionnaire in both the first and second administration showed significant positive correlation between functional limitations and malocclusion. The discriminant validity testing of the Hindi questionnaire also revealed a positive correlation between the total score and malocclusion (Tables 2 and 3).

Discriminant validity testing was also carried out with relation to overall and subscale scores for children with no malocclusion, with moderate malocclusion and severe malocclusion. The results revealed that the mean functional limitation scores were significantly higher in subjects with malocclusion than in subjects without mal-occlusion in both the English and Hindi questionnaires. Further, the independent sample “t” test brought out that the values of the mean total scores were significantly higher in the subjects who had malocclusion as compared with those without malocclusion in both versions of the questionnaires (Table 4). The construct validity was determined by drawing rank correlations between total scales, subscale scores, and comparing it with global ratings of oral health and overall well-being scores in both the versions of the questionnaires. However, there were no significant correlations between domain and total scores and global ratings scale (Tables 5 to 7). The reliability statistics for the translated Hindi version of the CPQ_11-14 _revealed a Cronbach’s alpha value of 0.852 for the total scale. The values ranged from 0.72 for oral symptoms, 0.77 for functional limitations, 0.85 for emotional well-being, 0.66 for social well-being, 0.82 for self-esteem, and 0.71 for psychological well-being. Thus, this brought out the fact that the Cronbach’s alpha value for reliability was acceptable and suggested good internal consistency and correlation among the items in the translated Hindi version of the questionnaire (Table 8).

**Table 1:** **Table 1:** Discriminant validity: rank correlations between DMFT, malocclusion (MO) index scores, and subscale scores (English)

				*DMFT*		*MO*	
OS_E		R		0.342*		0.415*	
		p-value		0.05		0.016	
FL_E		R		0.013		0.522**	
		p-value		0.943		0.002	
EW_E		R		0.021		0.336	
		p-value		0.905		0.056	
SW_E		R		0.101		0.334	
		p-value		0.574		0.057	
SE_E		R		0.167		0.311	
		p-value		0.352		0.079	
PW_E		R		–0.012		–0.191	
		p-value		0.949		0.287	
Total		R		0.199		0.544**	
		p-value		0.267		0.001	

**Correlation is significant at the 0.01 level (2-tailed); *Correlation is significant at the 0.05 level (2-tailed)

**Table 2:** **Table 2:** Discriminant validity: rank correlations between DMFT, malocclusion (MO) index scores, and subscale scores (Hindi 1)

				*DMFT*		*MO*	
OS_H1		R		0.226		0.191	
		p-value		0.206		0.286	
FL_H1		R		0.076		0.376*	
		p-value		0.673		0.031	
EW_H1		R		0.168		0.232	
		p-value		0.350		0.194	
SW_H1		R		–0.016		0.148	
		p-value		0.929		0.412	
SE_H1		R		0.117		0.227	
		p-value		0.518		0.204	
PW_H1		R		0.042		–0.056	
		p-value		0.818		0.758	
Total_H1		R		0.244		0.422*	
		p-value		0.171		0.014	

Correlation is significant at the 0.01 level (2-tailed); *Correlation is significant at the 0.05 level (2-tailed)

**Table 3:** **Table 3:** Discriminant validity: rank correlations between DMFT, malocclusion (MO) index scores, and subscale scores (Hindi 2)

				*DMFT*		*MO*	
OS_H2		R		0.140		0.125	
		p-value		0.438		0.487	
FL_H2		R		0.115		0.413*	
		p-value		0.524		0.017	
EW_H2		R		0.144		0.292	
		p-value		0.423		0.099	
SW_H2		R		–0.045		0.267	
		p-value		0.804		0.133	
SE_H2		R		0.103		0.336	
		p-value		0.570		0.056	
PW_H2		R		–0.042		–0.121	
		p-value		0.818		0.502	
Total_H2		R		0.163		0.485**	
		p-value		0.365		0.004	

**Correlation is significant at the 0.01 level; *Correlation is significant at the 0.05 level

**Table 4:** **Table 4:** Discriminant validity: overall scores for malocclusion with difference in means (English, Hindi 1, Hindi 2)

		*Malocclusion*									
		*No malocclusion (n = 7)*				*Moderate/severe (n = 26)*					
		*Mean*		*SD*		*Mean*		*SD*		*p-value*	
OS_E		4.43		1.40		6.88		3.44		0.077	
FL_E		2.86		2.73		9.04		6.89		0.001	
EW_E		5.71		3.86		9.54		6.99		0.177	
SW_E		3.43		2.15		6.00		5.87		0.268	
SE_E		5.86		3.80		10.65		7.27		0.105	
PW_E		36.43		3.87		36.42		5.67		0.998	
Total_E		58.71		6.21		78.54		21.16		<0.001	
OS_H1		4.57		2.15		6.62		3.80		0.185	
FL_H1		4.14		2.27		8.23		5.62		0.007	
EW_H1		6.43		3.69		9.12		6.62		0.176	
SW_H1		4.14		1.77		4.96		4.65		0.476	
SE_H1		5.71		4.15		9.15		7.06		0.23	
PW_H1		32.86		9.42		36.54		7.11		0.265	
Total_H1		57.86		8.97		74.62		19.88		0.004	
OS_H2		5.14		2.54		6.73		3.05		0.217	
FL_H2		4.00		2.08		8.62		5.19		0.001	
EW_H2		7.29		3.15		9.73		5.06		0.236	
SW_H2		3.86		1.46		5.65		4.40		0.3	
SE_H2		5.29		3.30		10.08		6.40		0.067	
PW_H2		33.14		7.06		35.15		7.20		0.515	
Total_H2		58.71		6.63		75.96		17.06		<0.001	

Independent sample “t” test; SD: Standard deviation

A paired t-test was also performed to check for test-retest reliability of the Hindi questionnaire. It showed that only social well-being scores showed significant difference between first and second time tested Hindi version questionnaires, while rest of the domains and total scores showed no significant differences between first and second time testing which suggested good reliability (Table 9).

**Table 5:** **Table 5:** Construct validity: rank correlations between total scale, subscale scores, global ratings of oral health and overall well-being (English)

				*Q1_E*		*Q2_E*	
OS_E		R		–0.050		0.107	
		p-value		0.782		0.552	
FL_E		R		–0.004		0.164	
		p-value		0.980		0.362	
EW_E		R		–0.063		0.198	
		p-value		0.727		0.270	
SW_E		R		–0.223		–0.163	
		p-value		0.212		0.363	
SE_E		R		–0.160		–0.067	
		p-value		0.373		0.710	
PW_E		R		0.127		0.071	
		p-value		0.483		0.693	
Total_E		R		–0.112		0.084	
		p-value		0.535		0.642	

**Table 6:** **Table 6:** Construct validity: rank correlations between total scale, subscale scores, global ratings of oral health and overall well-being (Hindi 1)

				*Q1_H1*		*Q2_H1*	
OS_H1		R		0.157		0.214	
		p-value		0.382		0.232	
FL_H1		R		0.067		0.184	
		p-value		0.709		0.307	
EW_H1		R		–0.167		–0.100	
		p-value		0.353		0.580	
SW_H1		R		–0.041		–0.029	
		p-value		0.821		0.871	
SE_H1		R		–0.087		–0.115	
		p-value		0.631		0.523	
PW_H1		R		0.048		–0.088	
		p-value		0.790		0.625	
Total_H1		R		0.009		–0.018	
		p-value		0.960		0.919	

**Table 7:** **Table 7:** Construct validity: rank correlations between total scale, subscale scores, global ratings of oral health and overall well-being (Hindi 2)

				*Q1_H2*		*Q2_H2*	
OS_H2		R		0.057		0.154	
		p-value		0.752		0.391	
FI_H2		R		0.034		0.070	
		p-value		0.849		0.697	
EW_H2		R		0.003		0.011	
		p-value		0.987		0.952	
SW_H2		R		–0.061		–0.134	
		p-value		0.736		0.458	
SE_H2		R		–0.106		–0.259	
		p-value		0.559		0.146	
PW_H2		R		0.161		0.085	
		p-value		0.370		0.639	
Total_H2		R		0.054		–0.041	
		p-value		0.767		0.821	

**Table 8:** **Table 8:** Reliability statistics for total scale and subscales

		*Number of items*		*Cronbach’s alpha*		*ICC (95% CI)*	
Total		62		0.852		0.744 (0.603-0.854)	
OS		6		0.728		0.668 (0.456-0.816)	
FL		9		0.775		0.744 (0.589-0.857)	
EW		9		0.854		0.849 (0.758-0.915)	
SW		8		0.667		0.656 (0.445-0.808)	
SE		14		0.82		0.813 (0.704-0.895)	
PW		16		0.714		0.518 (0.24-0.728)	

CI: Confidence interval; ICC, interclass correlation coefficient; OS, oral symptoms; FL, functional limit score; EW, emotional well-being score; SW, social well-being score; SE, self-esteem score; PW, psychological well-being score

**Table 9:** **Table 9:** Test-retest reliability of the Hindi questionnaire

		*H1*				*H2*					
		*Mean*		*SD*		*Mean*		*SD*		*p-value*	
OS		6.18		3.58		6.39		2.99		0.401	
FL		7.36		5.34		7.64		5.05		0.359	
EW		8.55		6.17		9.21		4.78		0.098	
SW		4.79		4.20		5.27		4.01		0.027; Sig	
SE		8.42		6.65		9.06		6.16		0.059	
PW		35.76		7.65		34.73		7.11		0.092	
Total		71.06		19.30		72.30		16.94		0.17	

Paired “t” test; SD: Standard deviation; OS, oral symptoms; FL, functional limit score; EW, emotional well-being score; SW, social well-being score; SE, self-esteem score; PW, psychological well-being score

## DISCUSSION

The study was conceived to formulate a tool to evaluate the perceptions of children in the 11- to 14-year-old age groups toward their oral health in general with major focus on DMFT and malocclusion, as these conditions are the most common among these children. The tool had to be understood by all the children and had to be socially and culturally relevant to the country where it had to be used.^14-17^ The CPQ_11-14_ is one such validated tool. This tool was, however, in English, which is a language alien to many children in India, especially those from rural backgrounds. The original CPQ_11-14_ was hence, modified by adding questions on self-esteem and psychological well-being and translated into Hindi. In the Hindi questionnaire, some of the questions had to be rephrased as the exact translation would not be culturally relevant. This also helped to increase its usability to assess the perceptions of children in India. The translated tool needed to be validated and checked for reliability prior to its wider use in the population. The children who visited our institute for the first time seeking orthodontic treatment in the age group of 11 to 14 years were administered the English questionnaire followed by the Hindi questionnaire to check for its validity. The same group of children was recalled after 1 week and administered the Hindi questionnaire as a retest to confirm reliability. The children who did not complete the questionnaires or did not fill the consent forms were not included in the final sample. The parents of the children were requested to refrain from participating in the process of filling the questionnaires in order to prevent influencing the perceptions of the child. Some of the children found the questionnaire to be too long; however, that did not seem to affect its reliability or validity as shown in the results. The addition of the domains of self-esteem and psychological well-being contributed to the increase in questions as compared with the original English questionnaire. However, this addition helped to better evaluate the oral health status of the child with its influence on newer parameters of QoL.

The various tests for reliability showed significant positive correlation between malocclusion and functional limitation in both the English and the Hindi questionnaires. There was significant positive correlation between oral symptoms, DMFT, and malocclusion in the English questionnaire; however, there was no correlation found between the DMFT scores and functional limitations in any of the questionnaires. This was in line with other such studies done in United Kingdom and Saudi Arabia.^18,19^ The evaluation of perceptions related to QoL associated with oral health is strongly influenced by personality and standards of reference; therefore, poor correlations between these domain scores may actually be not so unusual.^9,11^

The construct validity of the Hindi questionnaire was found to be not so significant in relation to the domain scores, total score, and global ratings. These were found to be significant in the Saudi and Canadian studies; however, the sample size used in those studies was far bigger and this may help in bringing out association between the domains in the construct validity. Further, it has been brought out in previous studies that global rating of health can vary with the race, culture, and education.^20^

The reliability of the questionnaire was evaluated by test-retest samples of the translated Hindi questionnaire. The test-retest reliability of the Hindi questionnaire was found to be good (0.17, p < 0.001) with significant differences found in the social well-being subscale. This could be because of the fact that the social well-being domain mainly dealt with the children’s oral health and its impact on their relations with other children in school. Since the second retest administration was after 1 week, the child’s perceptions of his relations to other children may have changed during the period.

The reliability was also evaluated using the Cronbach’s alpha test which showed substantial internal consistency and correlation among the items in the Hindi questionnaire (0.85, p < 0.001) which was similar to the Arabic (0.65, p < 0.001) and English (0.90, p < 0.001) language studies.^18,19^ The subscale scores were found to be satisfactory for validity when compared with the original English questionnaire; also the test-retest showed satisfactory reliability. The Hindi used in the translated version of the questionnaire is what is commonly taught to school children in India. As the spoken language may vary slightly with dialects used in different parts of the country, the written language is universal. This would help the applicability of the questionnaire in different parts of the country as was brought out with the studies done with the Arabic version in Syria, Egypt, and Saudi Arabia.^18^

One of the limitations of this study was in relation to the number of subjects; a larger group may have brought out more accurate results and will be attempted in the future. Further clinical examination was held with simple examination without the use of diagnostic tools like X-rays; therefore, dental diseases like dental caries were recorded only by visual examination. In the subsequent stage of the study, more accurate information will be recorded through more precise clinical diagnosis.

## CONCLUSION

The Hindi translation and modification of the CPQ_11-14 _with addition of new domains to record perceptions of children toward oral health showed acceptable validity and reliability in the sample who were investigated at our institute. This tool will be helpful in wider multicentric studies on children in different parts of the country and also help in cross-cultural comparison of perceptions related to oral health among children in India and other countries.
